# Chemical doping to control the in-situ formed doping structure in light-emitting electrochemical cells

**DOI:** 10.1038/s41598-023-38006-y

**Published:** 2023-07-15

**Authors:** Gunel Huseynova, Joan Ràfols-Ribé, Etienne Auroux, Ping Huang, Shi Tang, Christian Larsen, Ludvig Edman

**Affiliations:** 1 The Organic Photonics and Electronics Group, Department of Physics, Umeå University, 90187 Umeå, Sweden; 2 Department of Chemistry, Ångström Laboratory, Uppsala University, Box 523, 751 20 Uppsala, Sweden

**Keywords:** Lasers, LEDs and light sources, Organic LEDs, Nanocavities, Electrical and electronic engineering, Electronic devices

## Abstract

The initial operation of a light-emitting electrochemical cell (LEC) constitutes the in-situ formation of a p–n junction doping structure in the active material by electrochemical doping. It has been firmly established that the spatial position of the emissive p–n junction in the interelectrode gap has a profound influence on the LEC performance because of exciton quenching and microcavity effects. Hence, practical strategies for a control of the position of the p–n junction in LEC devices are highly desired. Here, we introduce a “chemical pre-doping” approach for the rational shifting of the p–n junction for improved performance. Specifically, we demonstrate, by combined experiments and simulations, that the addition of a strong chemical reductant termed “reduced benzyl viologen” to a common active-material ink during LEC fabrication results in a filling of deep electron traps and an associated shifting of the emissive p–n junction from the center of the active material towards the positive anode. We finally demonstrate that this chemical pre-doping approach can improve the emission efficiency and stability of a common LEC device.

Organic light-emitting diodes (OLEDs) can deliver highly efficient emission from thin-film (and flexible) device structures by virtue of a rational design of their constituent p–n junction doping structure^1^. This device design involves the careful inclusion of a multitude of different layers, some of which are chemically doped, during device fabrication, which enables for the localization of the emissive p–n junction at a point of constructive interference and for the suppression of exciton losses due to lowered quenching interactions with the electrodes, other excitons, and the dopants^2–4^.

The light-emitting electrochemical (LEC) is an alternative thin-film (and flexible) light-emission technology that is similar in appearance to the OLED, but which is distinguished by its much simpler device architecture that enables for cost-efficient ambient-air printing^5,6^ and coating^7,8^ fabrication. The LEC commonly comprises a single-layer active material sandwiched between two air-stabile electrodes^9–13^, and during operation the LEC-defining mobile ions in the active material redistribute for the formation of a p–n junction doping structure by electrochemical doping.^14–17^.

This characteristic *in-situ* electrochemical doping mode of LECs has the drawback that it has rendered the rational control of the p–n junction position in the interelectrode gap for high emission performance difficult. In this context, we call attention to that direct visual observations of open planar LEC devices with extremely large interelectrode gaps^18–20^ and angle-resolved electroluminescence (EL) measurements and simulations of thin-film LECs^21,22^ have showed that the selection of the mobile ions^23,24^, the ion transporter^25^, and the emissive organic semiconductor^26^ can have a profound influence on the position of the p–n junction. Recently, these experimental findings were rationalized by theoretical investigations that established that the initial position of the p–n junction in the interelectrode gap is determined by the anion/cation mobility ratio, while its steady-state position is decided by the electron/hole mobility ratio^25,27^. However, it is notable that these important measurement results and conceptual insights were invariably derived on LEC devices, which comprised a pristine and undoped organic semiconductor as the emissive and electrochemically active species.

In the present study, we demonstrate that a rational and controlled shift of the position of the emissive p–n junction in LEC devices can be attained by a new concept, viz. “chemical pre-doping” of the organic semiconductor already during the LEC fabrication. We specifically show that the addition of a soluble strong chemical reductant, in the form of the neutral organic molecule termed reduced benzyl viologen (r-BV^0^), to the active-material ink results in an electron transfer and an associated filling of deep electron traps of a common emissive organic semiconductor termed Super Yellow. By combined experimentation and simulation, we further show that this chemical pre-doping approach enables for a significant and rational spatial shift of the emissive p–n junction, that the magnitude of this shift can be controlled by the concentration of the chemical dopant, and that this addition can enable for an improved device performance in the form of an increased emission efficiency and stability.

## Results and discussion

**Figure 1:**
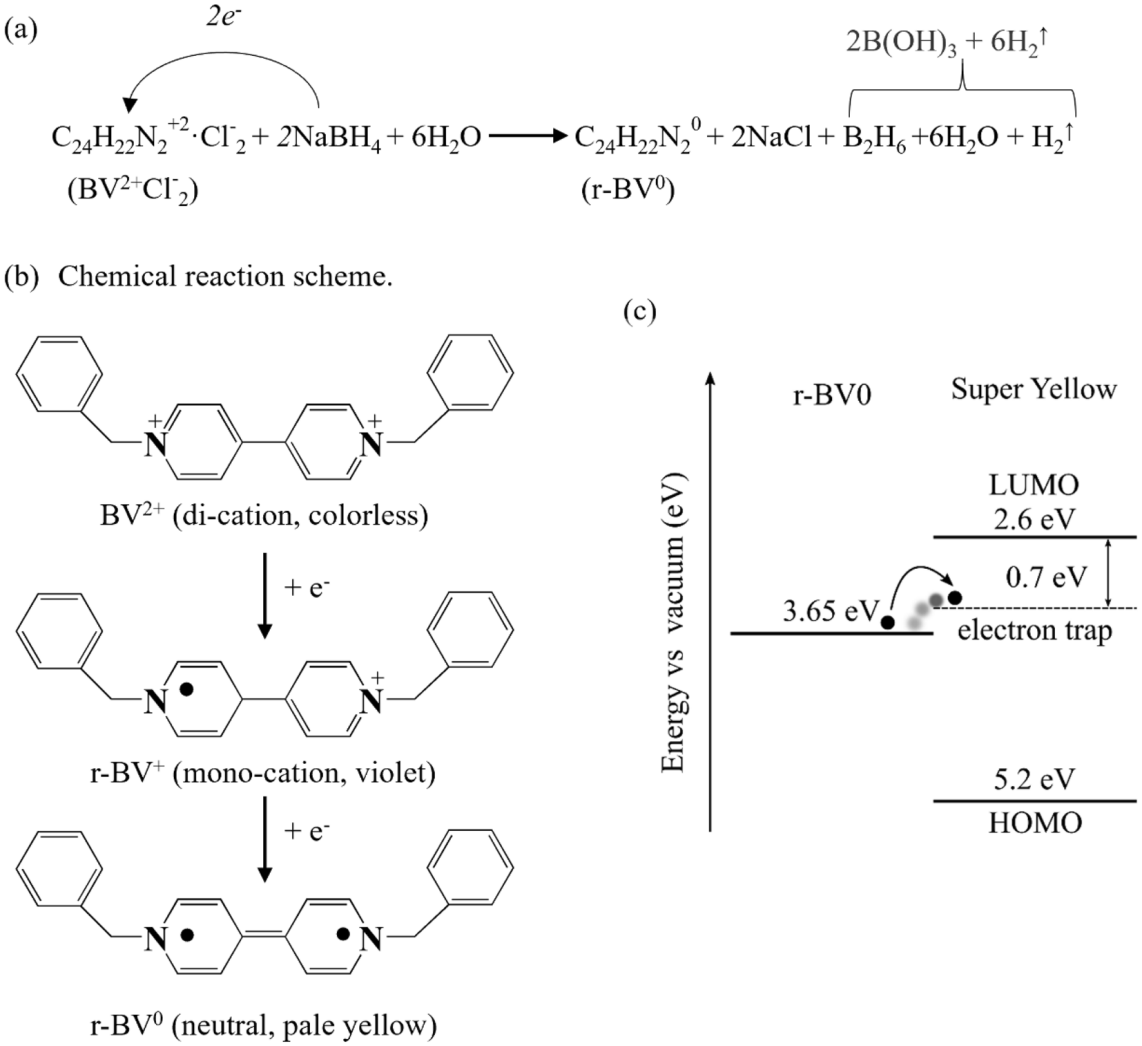
(**a**) The reaction scheme for the synthesis of the chemical reductant r-BV^0^. (**b**) The three different oxidation states of benzyl viologen, BV, with their corresponding color identified in the parenthesis. (**c**) The electron-energy diagram shows the HOMO of r-BV^0^ to the left, and the LUMO, the electron-trap level and the HOMO of the emissive organic semiconductor Super Yellow to the right. The arrow indicates an electron transfer from the HOMO of r-BV^0^ to the electron-trap level of Super Yellow.

Viologens are organic bipyridinium compounds that are widely studied and utilized for their electron-donating^28–30^, electrochromic^31^, and catalytic^32^, properties; in addition, their high solubility in common solvents render them compatible with cost-efficient printing and coating fabrication^28,33^. One member of the bipyridinium class is the neutral reduced benzyl viologen (r-BV^0^) molecule, which comprises a central 4,4′ − bipyridine core surrounded by two benzyl groups (see Figs. 1a,b). r-BV^0^ is a notably strong electron donor, *i.e.* chemical reductant^30^, which has been employed for the n-type doping of a wide variety of semiconductors, including conjugated polymers, carbon nanostructures, graphene, MoS_2_ and PbS^29,30,34–39^.

We herein synthesized r-BV^0^ by reducing a benzyl viologen dichloride salt (BV^2+^Cl^-^_2_) with the aid of a large surplus of a NaBH_4_ salt as the reducing agent^35–39^ in a two-component water:toluene solution, as schematically depicted in Fig. 1a. The exothermic reaction proceeds in two reduction steps, with the initial step featuring an electron transfer from NaBH_4_ to the nitrogen on one of the two pyridine rings of the BV^2+^ di-cation for the formation of a r-BV^+^ mono-cation; while the final step constitutes an electron transfer from NaBH_4_ to the nitrogen on the second of the pyridine rings for the formation of the desired neutral r-BV^0^ end product. The chemical structures of these three different oxidation states of the benzyl viologen (BV) molecule, and their associated colors, are displayed in Fig. 1b.

The progression of this chemical reaction can be observed visually, since the two reactants (BV^2+^Cl^-^_2_, and NaBH_4_) are colorless, while the r-BV^+^ intermediate is colored violet, and the final r-BV^0^ product is pale yellow^29,35,38–45^. The conclusion of the reaction can accordingly be determined by the reaction solution shifting color from violet to yellow (see Fig. S1), in combination with the disappearance of the H_2_ gas bubbles from the reaction solution (see Fig. 1a). The completion of this double-reduction reduction was further confirmed by the recording of a silent EPR spectrum (data not shown). The single-reduction product r-BV^+^ would in contrast have exhibited a characteristic EPR spectrum^46^. The employment of the poorly miscible two-component water:toluene reaction solution enabled for the practical collection of the neutral r-BV^0^ product from the upper (and lighter) toluene phase, since both reactants and all other non-gaseous products are solely soluble in the lower water phase. More details on the synthesis are available in the Methods section.

The extracted “doping solution” (*i.e.*, r-BV^0^ dissolved in toluene) exhibited a r-BV^0^ solute concentration of ~ 4 g·l^−1^. This solution was used as the chemical reductant in the formulation of the ”active-material inks”, which also comprised the electroluminescent and polymeric organic semiconductor termed Super Yellow, the ion transporter hydroxyl-capped trimethylolpropane ethoxylate (TMPE-OH), and the salt KCF_3_SO_3_ in a constant mass ratio of 1:0.1:0.03. The r-BV^0^ concentration was varied in the different inks, as quantified by the mass fraction of r-BV^0^ with respect to Super Yellow.

Figure 1c is an electron-energy diagram, which presents values for the HOMO of r-BV^0^ to the left and the LUMO, HOMO and an electron-trap level of Super Yellow^47–49^ to the right. The HOMO value for r-BV^0^ was derived from a cyclic voltammetry (CV) measurement *vs.* the standard hydrogen electrode (SHE), and by using the convention that the SHE electrode is offset by 4.44 eV from the vacuum level.^30,50,51^ The HOMO and LUMO of Super Yellow were similarly determined from CV measurements,^52^ while a major and generic electron trap level in organic semiconductors was identified and rationalized by Blom et al.^47,49^ A recent LEC study further suggest that the inclusion of the TMPE-OH ion transporter into the active material of our LEC devices can result in the formation of a significant concentration of additional electron traps^25^, but their exact energy level is currently unknown. Nevertheless, the presented electron-energy diagram implies that an electron transfer from the HOMO of r-BV^0^ to the established electron-trap level of Super Yellow should be possible, as indicated by the arrow in Fig. 1c.

**Figure 2:**
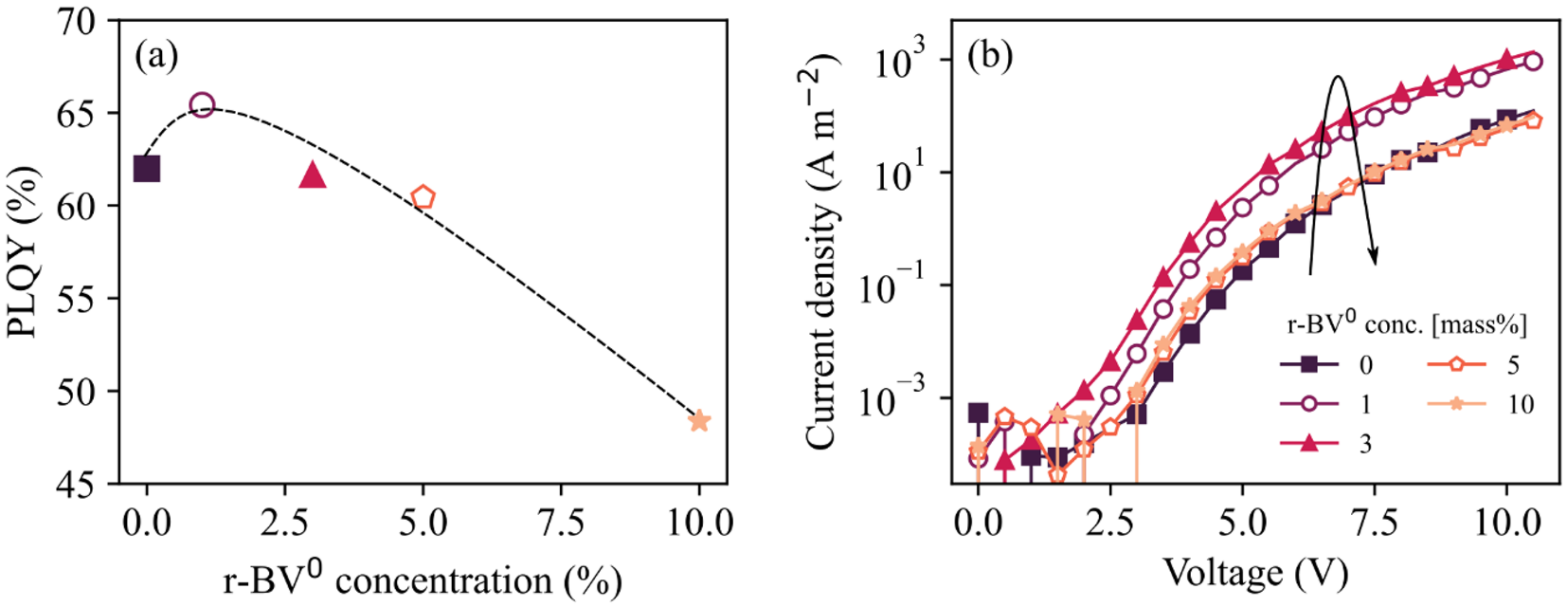
(**a**) The PLQY of the (Super Yellow + TMPE-OH + KCF_3_SO_3_ + r-BV^0^) active material as a function of the r-BV^0^ chemical reductant concentration. The dashed line is a guide to the eye. (**b**) The current density as a function of the applied voltage for glass/ITO/Al/(Super Yellow + r-BV^0^)/Ca/Al electron-only devices for different concentrations of the r-BV^0^ chemical reductant, as specified in the legend. The thickness of the active material is 100 nm.

The photoluminescence quantum yield (PLQY) is a sensitive indicator of the existence of “trap impurities” in organic semiconductors, since the singlet exciton typically diffuses a significant distance of ~ 10 nm during its lifetime in neat organic semiconductors^53^, and since the exciton commonly is efficiently quenched by such trap impurities^54,55^. Figure 2a presents the evolution of the PLQY of the active-material film as a function of the added r-BV^0^ concentration. Interestingly, we find that the PLQY first increases up to ~ 2 mass% of r-BV^0^, and thereafter decreases monotonously with further increasing r-BV^0^ concentration. The initial increase of the PLQY implies that the first added r-BV^0^ molecules effectively eliminates “dark” electron traps, presumably by donating electrons and thereby filling of electron trap levels, as schematically depicted in Fig. 1c.

The influence on the electronic conductivity of Super Yellow by the addition of r-BV^0^ was investigated by the fabrication and characterization of glass/ITO/Al/(Super Yellow + r-BV^0^)/Ca/Al electron-only devices. Figure 2b shows that the current density increases by more than one order of magnitude over the entire probed voltage interval when the r-BV^0^ concentration is increased from 0 to 3 mass%; but that trend is reversed at higher r-BV^0^ concentrations exceeding 3 mass%, for which the electronic conductivity is reverting back to the lower level of intrinsic Super Yellow.

Accordingly, the summary conclusion from the data presented in Figs. 1c and S1 is that the first addition of r-BV^0^ molecules (up to 2–3 mass%) results in a filling of electron traps of Super Yellow, which is manifested in an increased electron mobility (and thereby an improved electronic conductivity)^56^ and an improved PLQY. A higher concentration of added r-BV^0^ molecules results in transport- and emission-damaging effects that obviously should be avoided in devices. We now turn our attention to the investigation of the effects of this dopant-enabled electron-trap filling approach on the performance of LEC devices.

**Figure 3:**
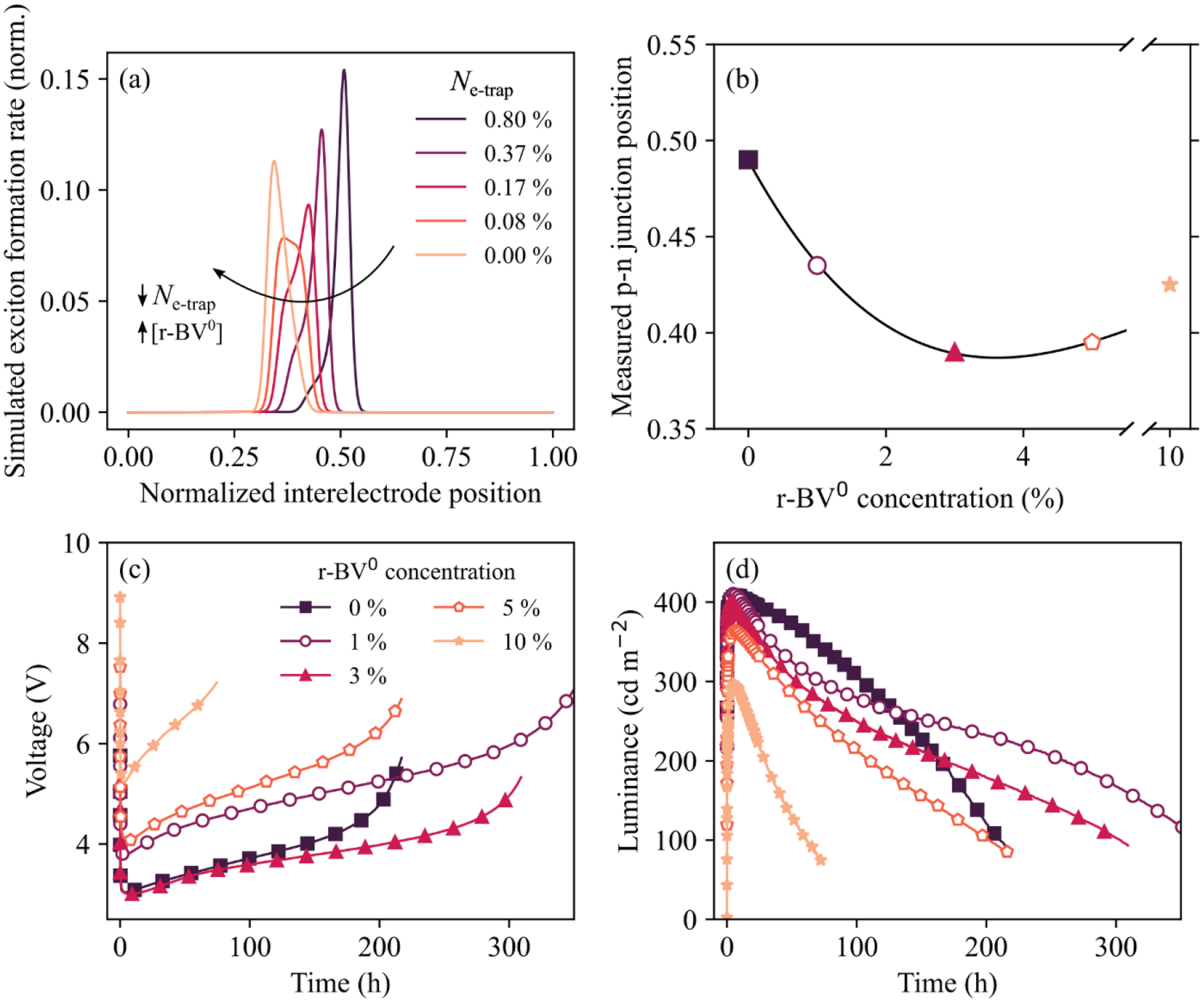
(**a**) The simulated position of the emissive p–n junction in the interelectrode gap for different values of the electron-trap concentration, as identified in the inset. The positive anode is located at 0 and the negative cathode at 1. The electron-trap concentration is normalized by the total concentration of transport states and expressed as a percentage in the inset. The arrow indicates the simulated anodic shift of the p–n junction with decreasing concentration of electron traps, i.e., an increasing number of chemical-reductant molecules. (**b**) The measured steady-state position of the emissive p–n junction as a function of the concentration of the r-BV^0^ chemical reductant. (**c**, **d**) The long-term temporal evolution of (**c**) the voltage and (**d**) the luminance during electrical driving of ITO/(Super Yellow + TMPE-OH + KCF_3_SO_3_ + r-BV^0^)/Al LECs by a constant current density of 7.75 mA·cm^-2^. The concentration of the chemical reductant r-BV^0^ is identified in the inset in (**c**).

Figure 3a presents the simulated steady-state exciton profile in the interelectrode gap for ITO/active-material/Al LECs, which are solely distinguished by the non-filled electron-trap concentration on the organic semiconductor in the active material, as identified in the inset. The trap-free electron/hole mobility ratio was set to 3 in the simulation, and the anodic (cathodic) interface is positioned at a normalized interelectrode position of 0 (1). The peak exciton formation rate can be considered to represent the position of the emissive p–n junction.

The simulation shows that the position of the p–n junction shifts from the center of the active material towards the positive anode with decreasing concentration of electron traps, *i.e.,* with increasing concentration of chemical-reductant molecules, as highlighted by the arrow. This behavior is explained by that the steady-state position of the p–n junction in LEC devices is determined by the effective electron/hole mobility ratio, with a higher electron (hole) mobility resulting in a p–n junction positioned closer to the positive anode (negative cathode). The filling of electron traps will increase the effective electron mobility, and it is accordingly expected to result in the observed shift of the p–n junction towards the positive anode.

We have experimentally investigated the effects of the addition of the r-BV^0^ chemical reductant on the p–n junction position by first measuring the angle-dependent EL spectrum and intensity for ITO/(Super Yellow + TMPE-OH + KCF_3_SO_3_ + r-BV^0^)/Al LECs and by thereafter simulating the same data. The sole free parameter in the simulation was the position of the emissive p–n junction, and by systematically determining the value for this parameter that produced the best agreement between the measured and simulated data, we could establish the position of emissive p–n junction with high accuracy^25,57,58^. A number of visual examples of this best-fit procedure for the identification of the p–n junction position are displayed in Fig. S3, while the Methods section provides more details on its practical execution.

Figure 3b presents the experimentally determined steady-state position of the p–n junction in the interelectrode gap as a function of the r-BV^0^ concentration. The LEC device void of r-BV^0^ exhibits a steady-state p–n junction in the exact middle of the active material at 0.50, while the addition of the r-BV^0^ chemical reductant to the active material results in a gradual and significant anodic shift of the p–n junction position to 0.43 at [r-BV^0^] = 1 mass% and 0.39 at [r-BV^0^] = 3 mass%. This experimental finding is thus in excellent agreement with the simulation results displayed in Fig. 3a. At a higher r-BV^0^ concentration ([r-BV^0^] ≥ 5 mass%) the anodic shift is halted, which is in line with our earlier finding that these higher r-BV^0^ concentrations cause transport (and emission) limiting effects.

We have finally investigated the effects of this chemical pre-doping approach, and its induced p–n junction shift, on the performance of LEC devices. Figure 3c,d display the measured voltage and luminance transients, respectively, of representative ITO/(Super Yellow + TMPE-OH + KCF_3_SO_3_ + r-BV^0^)/Al LEC devices during electrical driving by a constant current density of 7.75 mA·cm^-2^. The r-BV^0^ concentration is identified in the inset of Fig. 3c. The corresponding shorter-time luminance and current-efficacy transients are displayed in Fig. S4. We have measured eight independent devices for each r-BV^0^ concentration, and Fig. S2 and Table S1 present the average and standard deviation for key device metrics.

All investigated LEC devices feature a decreasing voltage and an increasing luminance during the initial operation, and a fast turn of < 2 s to a luminance exceeding 100 cd·m^−2^. This implies that the LEC-characteristic in-situ electrochemical doping capacity of the organic semiconductor Super Yellow is not significantly damaged or affected by the addition of r-BV^0^^59^. We find that the LEC devices with a r-BV^0^ concentration of ≤ 3 mass% exhibit a markedly better performance than the LECs with a higher r-BV^0^ concentration (see also Fig. S2 and Table S1). This supports our earlier finding that a too high r-BV^0^ concentration causes transport and emission limiting effects within the active material.

Importantly, we find that the LEC with 1 mass% r-BV^0^ exhibits ~ 10% higher peak luminance and ~ 70% longer operational lifetime than the reference LEC void of r-BV^0^, as gleaned from the statistical analysis presented in Table S1. (The operational lifetime is here defined as the total time that the device emits with a luminance exceeding 100 cd·m^−2^). We assign these improvements to the combined effects of a slightly increased PLQY (Fig. 2a), and the associated suppression of weakly emissive trap-assisted electron and hole recombination^49^, in combination with the anodic shift of the emissive p–n junction following the addition of 1 mass% r-BV^0^ to the active material (Fig. 3b). An anodic shift of the exciton population can for this particular device be attractive from an emission-efficiency viewpoint since the ITO anode is a less potent exciton quencher than the Al cathode^60,61^. Accordingly, a shift of the emissive p–n junction from the center of the active material towards the positive ITO anode can be expected to result in lowered losses due to exciton-electrode quenching. We finally note that exciton quenching can result in severe self heating^62,63^ and/or the formation of highly localized high-energy species on the organic semiconductor, which in turn can cause material and device degradation. Thus, the suppression of exciton quenching reactions by the electron trap filling of the organic semiconductor by the chemical reductant can also rationalize the observed prolongation of the device lifetime.

## Conclusions

We report on chemical pre-doping as a novel and functional tool for the rational adjustment of the doping structure and the p–n junction position in LEC devices. Such a tool is much desired, since it is well established that the in situ formed doping structure has a strong influence on the LEC performance. We specifically synthesize a solution-processible r-BV^0^ chemical reductant with a high HOMO level, which enables for effective electron transfer to a generic electron trap level of organic semiconductors. By a combination of simulations and experiments, we show that the addition of this chemical reductant to the active material of a common LEC device results in a shift of the emissive p–n junction from the center of the active material towards the transparent positive anode, and that such an optimized addition can result in a markedly improved device performance.

## Methods

### Synthesis of the chemical reductant

The overall reaction scheme for the chemical synthesis of the reduced benzyl viologen (r-BV^0^) chemical reductant is displayed in Fig. 1a. The reaction is initiated by mixing 20 mg (0.05 mmol) benzyl viologen dichloride salt (BV^2+^Cl^−^_2_, C_24_H_22_N_2_^2+^Cl^−^_2_, *M*_W_ = 409.35 g·mol^−1^, 97%, Merck, GER) with 4 g (0.1 mol) NaBH_4_ salt (*M*_W_ = 37.83 g·mol^−1^, powder, ≥ 98.0%, Merck, GER) in 10 ml (0.6 mol) deionized water, followed by the addition of 5 ml (0.05 mol) of toluene. The B_2_H_6_ product is quickly reacting with water to form B(OH)_3_ boric acid and H_2_ gas, with the latter being observed as gas bubbles escaping the reaction liquid.

At the end of the reaction, the water and toluene solvents are visibly separated, with the lower-density toluene floating on top of the water. Both reactants (*i.e.* the two salts BV^2+^Cl^-^_2_ and NaBH_4_) and all non-gaseous products are highly soluble in water but effectively insoluble in toluene^28^, with the exception being the neutral r-BV^0^ reaction product that instead is highly soluble in toluene^29,35,38–41^. This distinct dissolution capacity enables for the facile collection of r-BV^0^ at the end of the reaction through the extraction of the lighter toluene phase with a syringe.

Figures 1b and S1 reveal that the progression of the reaction can be visualized through the change in color that accompanies the two-step reduction of the BV molecule. The original BV^2+^ di-cation reactant is colorless, while the intermediate r-BV^+^ mono-cation is violet, and the final neutral r-BV^0^ product is pale yellow in color. We could thus determine the conclusion of the reaction by the time at which the toluene phase had changed color to yellow and when the formation of H_2_ gas bubbles had ceased. The time for complete reaction was approximately three days. It is notable that the reaction was executed under ambient air at room temperature without the use of a catalyst. The r-BV^0^ concentration in the extracted 5 ml toluene solution is ~ 4 g·l^−1^, and from here on we refer to it as the "doping solution".

### Ink formulation

The electroluminescent organic semiconductor Super Yellow (PDY-132, Merck, GER) was dissolved in cyclohexanone (purity > 99.5%, Sigma Aldrich, USA) in a concentration of either 8 or 10 g·l^−1^. The KCF_3_SO_3_ (Merck, GER) salt and the hydroxyl-capped trimethylolpropane ethoxylate (TMPE-OH, *M*_W_ = 450 g·mol^−1^, Merck, GER) ion transporter were separately dissolved in cyclohexanone in a 10 g·l^−1^ concentration. KCF_3_SO_3_ and TMPE-OH were dried under vacuum at 80 °C for 12 h before dissolution, while the other chemicals were used as received.

The master solutions were stirred on a magnetic hot plate at 70 °C for 1 day, and thereafter blended in a Super Yellow:TMPE-OH:KCF_3_SO_3_ mass ratio of 1:0.1:0.03. The doping solution was thereafter added in a number of different concentrations for the formulation of the active-material inks. These inks are solely distinguished by the concentration of r-BV^0^, which is herein quantified by the mass ratio between r-BV^0^ and Super Yellow. The inks for the electron-transport measurements were prepared in an identical manner, but with the TMPE-OH and KCF_3_SO_3_ master solutions excluded. All of the ink preparation and device fabrication processes were performed in a pair of interconnected N_2_-filled glove boxes ([O_2_] < 1 ppm, [H_2_O] < 1 ppm).

### Film and device fabrication and characterization

The thin films for the optical characterization were spin-coated onto carefully cleaned quartz substrates using the inks based on the lower viscosity 8 g·l^−1^ Super Yellow master solution. The employment of a spin speed of 1700–2000 rpm for 60 s resulted in a ~ 110 nm dry film thickness, as determined by a stylus profilometer (Dektak XT, Bruker, US). The photoluminescence quantum yield (PLQY) was measured with an integrating sphere connected to a multi-channel spectrometer (C9920-02G, Hamamatsu Photonics, JP).

For the LEC devices, pre-patterned indium-tin oxide (ITO) coated glass substrates (ITO thickness = 145 nm, ITO sheet resistance = 20 Ω/sq, substrate area = 30 × 30 mm^2^, Kintech, CHN) were cleaned by sequential 15 min ultrasonic treatment in detergent (Extran MA 01, Merck, GER), deionized water (two cycles to completely remove detergent), acetone, and isopropanol, and then dried in an oven at 120 °C for > 30 min. The ITO substrates were treated by a UV/ozone treatment for 10 min prior to spin coating to improve the ink wettability.

For the angle-dependent LEC measurement, the active-material ink based on the higher viscosity 10 g·l^−1^ Super Yellow master solution was spin coated with a spin speed of 2000–2300 rpm for 120 s, for the attainment of a dry active-material film thickness of ~ 175 nm. For the forward-luminance LEC measurement, the active-material ink based on the lower-viscosity 8 g·l^−1^ Super Yellow master solution was spin coated at a spin speed of 1500–2000 rpm for 60 s, for the attainment of a dry active-material film thickness of ~ 110 nm. The spin-coated active-material films were dried at 70 °C for 1 h on a hot plate.

The 100-nm thick Al top electrodes were deposited by thermal evaporation in a vacuum chamber (*p* ≤ 5 × 10^–4^ Pa), with a quartz crystal monitoring the Al evaporation rate (5–7 Å·s^-1^) and the Al thickness. A shadow mask positioned in between the evaporator source and the LEC device defined the size and shape of the Al electrodes. The spatial overlap between the Al and ITO electrodes defined four 2 × 2 mm^2^ LEC devices on each substrate. The Al top electrode was invariably biased as the negative cathode in all measurements.

The electron-only glass/ITO/Al/(Super Yellow:r-BV^0^)/Ca/Al devices were fabricated in a similar manner, but with a 100 nm thick Al electrode thermally evaporated onto the ITO surface, a 100 nm thick Super Yellow:r-BV^0^ blend film spin-coated onto the Al, and a 20 nm thick Ca layer thermally evaporated on the blend film.

The forward-luminance and the electron-only measurements were performed on non-encapsulated devices in the glove box. The forward luminance and the voltage were measured at a constant current density of 7.75 mA·cm^−2^, using a computer-controlled OLED lifetime setup (M6000 PMX, McScience, KOR).

The angle-dependent measurements were conducted under ambient air, and these devices were therefore encapsulated by attaching a 24 × 24 mm^2^ glass cover slide (Menzel GmbH, GER) onto the Al cathode side with UV-curable epoxy (E132-60 mL, Ossila). The epoxy was cured by exposure to UV light (*λ*_peak_ = 365 nm, power density = 80 mW mm^−2^, UV-Exposure Box 1, Gie-Tec) for 15 min. The angle-dependent EL measurements were performed with a custom-built spectrogoniometer setup, essentially comprising a rotation stage, a stepper motor, and a fiber-optic CCD-array spectrometer (Flame-S, Ocean Optics)^22^. The forward emission was measured at 0°, and the viewing angle was varied from −80° to 80° in 10° steps. The spectrogoniometer was controlled with a Python-based virtual instrument using a Raspberry Pi 400. For these measurements, the devices were driven by a constant current density of 25 mA·cm^-2^ at a 21 V compliance voltage, with the current supplied and the voltage measured with a source measure unit (Keithley 2400).

The optical simulations were carried out with a commercial software (Setfos 5.2, Fluxim AG, CHE). The position of the emissive p–n junction within the active material was determined by minimizing the root mean square error between the simulated and the measured angle-dependent EL data. Further details on this procedure can be found in Ref^22^. The exciton formation rate profile in the interelectrode gap was determined with the drift–diffusion module of the same software (Setfos 5.3, Fluxim AG, CHE), and the simulated three-layer device featured an ITO anode (thickness = 145 nm), an active material (thickness = 150 nm), and an Al cathode. The parameter values for this device structure were gleaned from literature references^25,64^ and are listed in Table S1 in the Supporting Information. The simulated devices were driven by a constant voltage of 3 V.

## Supplementary Information

Supplementary Information.

## Data Availability

The data that support the findings of this study are available from the corresponding author upon reasonable request.

## References

[1] Reineke S, Lindner F, Schwartz G, Seidler N, Walzer K, Lüssem B, Leo K (2009). White organic light-emitting diodes with fluorescent tube efficiency. Nature.

[2] Friend RH, Gymer RW, Holmes AB, Burroughes JH, Marks RN, Taliani C, Bradley DDC, Santos DAD, Brédas JL, Lögdlund M, Salaneck WR (1999). Electroluminescence in conjugated polymers. Nature.

[3] Shin JH, Matyba P, Robinson ND, Edman L (2007). The influence of electrodes on the performance of light-emitting electrochemical cells. Electrochim. Acta.

[4] Rörich I, Niu Q, van der Zee B, del Pino Rosendo E, Crăciun NI, Ramanan C, Blom PWM (2020). Exciton ses. Advanced Electronic Materials.

[5] Hernandez-Sosa G, Tekoglu S, Stolz S, Eckstein R, Teusch C, Trapp J, Lemmer U, Hamburger M, Mechau N (2014). The compromises of printing organic electronics: A case study of gravure-printed light-emitting electrochemical cells. Adv. Mater..

[6] Lindh EM, Sandström A, Edman L (2014). Inkjet printed bilayer light-emitting electrochemical cells for display and lighting applications. Small.

[7] Sandström A, Dam HF, Krebs FC, Edman L (2012). Ambient fabrication of flexible and large-area organic light-emitting devices using slot-die coating. Nat. Commun..

[8] Sandström A, Asadpoordarvish A, Enevold J, Edman L (2014). Spraying light: Ambient-air fabrication of large-area emissive devices on complex-shaped surfaces. Adv. Mater..

[9] Mishra A, DiLuzio S, Alahbakhshi M, Adams AC, Bowler MH, Moon J, Gu Q, Zakhidov AA, Bernhard S, Slinker JD (2021). Bright single-layer perovskite host-ionic guest light-emitting electrochemical cells. Chem. Mater..

[10] Liu C-Y, Bard AJ (2002). individually addressable submicron scale light-emitting devices based on electroluminescence of solid Ru(bpy)_3_(ClO_4_)_2_ films. J. Am. Chem. Soc..

[11] Slinker JD, Gorodetsky AA, Lowry MS, Wang J, Parker S, Rohl R, Bernhard S, Malliaras GG (2004). Efficient yellow electroluminescence from a single layer of a cyclometalated iridium complex. J. Am. Chem. Soc..

[12] Maness KM, Terrill RH, Meyer TJ, Murray RW, Wightman RM (1996). Solid-state diode-like chemiluminescence based on serial, immobilized concentration gradients in mixed-valent Poly[Ru(vbpy)_3_](PF_6_)_2_ Films. J. Am. Chem. Soc..

[13] Alahbakhshi M, Mishra A, Haroldson R, Ishteev A, Moon J, Gu Q, Slinker JD, Zakhidov AA (2019). Bright and effectual perovskite light-emitting electrochemical cells leveraging ionic additives. ACS Energy Lett..

[14] Pei QB, Yu G, Zhang C, Yang Y, Heeger AJ (1995). Polymer light-emitting electrochemical-cells. Science.

[15] Matyba P, Maturova K, Kemerink M, Robinson ND, Edman L (2009). The dynamic organic p–n junction. Nat. Mater..

[16] Meier SB, van Reenen S, Lefevre B, Hartmann D, Bolink HJ, Winnacker A, Sarfert W, Kemerink M (2013). Dynamic doping in planar ionic transition metal complex-based light-emitting electrochemical cells. Adv. Func. Mater..

[17] Fresta E, Weber MD, Fernandez-Cestau J, Costa RD (2019). White light-emitting electrochemical cells based on deep-red Cu(I) complexes. Adv. Opt. Mater..

[18] Gao J, Dane J (2003). Planar polymer light-emitting electrochemical cells with extremely large interelectrode spacing. Appl. Phys. Lett..

[19] Gao J, Dane J (2004). Visualization of electrochemical doping and light-emitting junction formation in conjugated polymer films. Appl. Phys. Lett..

[20] Shin JH, Dzwilewski A, Iwasiewicz A, Xiao S, Fransson A, Ankah GN, Edman L (2006). Light emission at 5 V from a polymer device with a millimeter-sized interelectrode gap. Appl. Phys. Lett..

[21] Su H-C (2018). Optical techniques for light-emitting electrochemical cells. ChemPlusChem.

[22] Lindh EM, Lundberg P, Lanz T, Edman L (2019). Optical analysis of light-emitting electrochemical cells. Sci. Rep..

[23] Shin JH, Robinson ND, Xiao S, Edman L (2007). Polymer light-emitting electrochemical cells: Doping concentration, emission-zone position, and turn-on time. Adv. Func. Mater..

[24] Hu YF, Gao J (2006). Cationic effects in polymer light-emitting electrochemical cells. Appl. Phys. Lett..

[25] Ràfols-Ribé J, Zhang X, Larsen C, Lundberg P, Lindh EM, Mai CT, Mindemark J, Gracia-Espino E, Edman L (2022). Controlling the emission zone by additives for improved light-emitting electrochemical cells. Adv. Mater..

[26] Matyba P, Andersson MR, Edman L (2008). On the desired properties of a conjugated polymer-electrolyte blend in a light-emitting electrochemical cell. Org. Electron..

[27] Diethelm M, Schiller A, Kawecki M, Devižis A, Blülle B, Jenatsch S, Knapp E, Grossmann Q, Ruhstaller B, Nüesch F, Hany R (2020). The dynamic emission zone in sandwich polymer light-emitting electrochemical cells. Adv. Func. Mater..

[28] Kathiresan M, Ambrose B, Angulakshmi N, Mathew DE, Sujatha D, Stephan AM (2021). Viologens: a versatile organic molecule for energy storage applications. J. Mater. Chem. A.

[29] Kim CS, Lee S, Tinker LL, Bernhard S, Loo Y-L (2009). Cobaltocene-doped viologen as functional components in organic electronics. Chem. Mater..

[30] Kiriya D, Tosun M, Zhao P, Kang JS, Javey A (2014). Air-stable surface charge transfer doping of MoS_2_ by benzyl viologen. J. Am. Chem. Soc..

[31] Shah KW, Wang S-X, Soo DXY, Xu J (2019). Viologen-based electrochromic materials: From small molecules, polymers and composites to their applications. Polymers-Basel.

[32] Hou S, Chen N, Zhang P, Dai S (2019). Heterogeneous viologen catalysts for metal-free and selective oxidations. Green Chem..

[33] Park JY, Lee SB, Park YS, Park YW, Lee CH, Lee JI, Shim HK (1998). Doping effect of viologen on photoconductive device made of poly (p-phenylenevinylene). Appl. Phys. Lett..

[34] Yu WJ, Liao L, Chae SH, Lee YH, Duan X (2011). Toward tunable band gap and tunable dirac point in bilayer graphene with molecular doping. Nano Lett..

[35] Kim SM, Jang JH, Kim KK, Park HK, Bae JJ, Yu WJ, Lee IH, Kim G, Loc DD, Kim UJ, Lee E-H, Shin H-J, Choi J-Y, Lee YH (2009). Reduction-controlled viologen in bisolvent as an environmentally stable n-type dopant for carbon nanotubes. J. Am. Chem. Soc..

[36] Jo K, Choi J, Kim H (2017). Benzyl viologen-assisted simultaneous exfoliation and n-doping of MoS_2_ nanosheets via a solution process. J. Mater. Chem. C.

[37] Nugraha MI, Kumagai S, Watanabe S, Sytnyk M, Heiss W, Loi MA, Takeya J (2017). Enabling ambipolar to heavy n-type transport in PbS quantum dot solids through doping with organic molecules. ACS Appl. Mater. Interfaces..

[38] Huseynova G, Shrestha NK, Xu Y, Shin E-Y, Park W-T, Ji D, Noh Y-Y (2018). Benzyl viologen as an n-type dopant for organic semiconductors. Org. Electron..

[39] Fukui A, Miura K, Ichimiya H, Tsurusaki A, Kariya K, Yoshimura T, Ashida A, Fujimura N, Kiriya D (2018). Reaction of N, N’-dimethylformamide and divalent viologen molecule to generate an organic dopant for molybdenum disulfide. AIP Adv..

[40] Striepe L, Baumgartner T (2017). Viologens and their application as functional materials. Chem. Eur. J..

[41] Bird CL, Kuhn AT (1981). Electrochemistry of the viologens. Chem. Soc. Rev..

[42] Rawashdeh AM, Ata BMB, Marji D, Mizyed S (2018). Complexation between viologens and some macrocyclic molecules: A cyclic voltammetry study. Jordan J. Chem..

[43] Ding J, Zheng C, Wang L, Lu C, Zhang B, Chen Y, Li M, Zhai G, Zhuang X (2019). Viologen-inspired functional materials: synthetic strategies and applications. J. Mater. Chem. A.

[44] Cao L, Fang G, Wang Y (2017). Electroreduction of viologen phenyl diazonium salts as a strategy to control viologen coverage on electrodes. Langmuir.

[45] Berville M, Karmazin L, Wytko JA, Weiss J (2015). Viologen cyclophanes: Redox controlled host–guest interactions. Chem. Commun..

[46] Lapkowski M, Bidan G (1993). Electrochemical, spectroelectrochemical and EPR properties of poly(pyrrole-viologens). J. Electroanal. Chem..

[47] Kuik M, Wetzelaer G-JAH, Nicolai HT, Craciun NI, De Leeuw DM, Blom PWM (2014). 25th anniversary article: Charge transport and recombination in polymer light-emitting diodes. Adv. Mater..

[48] Diethelm M, Bauer M, Hu W-H, Vael C, Jenatsch S, Blom PWM, Nüesch F, Hany R (2022). Electron trap dynamics in polymer light-emitting diodes. Adv. Func. Mater..

[49] Abbaszadeh D, Kunz A, Kotadiya NB, Mondal A, Andrienko D, Michels JJ, Wetzelaer G-JAH, Blom PWM (2019). Electron trapping in conjugated polymers. Chem. Mater..

[50] Dexter Tam TL, Lin TT, Omer MI, Wang X, Xu J (2020). The benzyl viologen radical cation: an effective n-dopant for poly(naphthalenediimide-bithiophene). J. Mater. Chem. A.

[51] Trasatti S (1986). The absolute electrode potential: An explanatory note (Recommendations 1986). Pure Appl. Chem..

[52] Sandström A, Matyba P, Edman L (2010). Yellow-green light-emitting electrochemical cells with long lifetime and high efficiency. Appl. Phys. Lett..

[53] Rörich I, Schönbein A-K, Mangalore DK, Halda Ribeiro A, Kasparek C, Bauer C, Crăciun NI, Blom PWM, Ramanan C (2018). Temperature dependence of the photo- and electroluminescence of poly(p-phenylene vinylene) based polymers. J. Mater. Chem. C.

[54] Tang S, Sandström A, Lundberg P, Lanz T, Larsen C, van Reenen S, Kemerink M, Edman L (2017). Design rules for light-emitting electrochemical cells delivering bright luminance at 27.5 percent external quantum efficiency. Nat. Commun..

[55] van Reenen S, Kersten SP, Wouters SHW, Cox M, Janssen P, Koopmans B, Bobbert PA, Kemerink M (2013). Large magnetic field effects in electrochemically doped organic light-emitting diodes. Phys. Rev. B.

[56] Kotadiya NB, Mondal A, Blom PWM, Andrienko D, Wetzelaer G-JAH (2019). A window to trap-free charge transport in organic semiconducting thin films. Nat. Mater..

[57] Lin G-R, Chen H-F, Shih H-C, Hsu J-H, Chang Y, Chiu C-H, Cheng C-Y, Yeh Y-S, Su H-C, Wong K-T (2015). Non-doped solid-state white light-emitting electrochemical cells employing the microcavity effect. Phys. Chem. Chem. Phys..

[58] Wang TW, Su HC (2013). Extracting evolution of recombination zone position in sandwiched solid-state light-emitting electrochemical cells by employing microcavity effect. Org. Electron..

[59] Tang S, Buchholz HA, Edman L (2015). On the selection of a host compound for efficient host-guest light-emitting electrochemical cells. J. Mater. Chem. C.

[60] Lanz T, Lindh EM, Edman L (2017). On the asymmetric evolution of the optical properties of a conjugated polymer during electrochemical p- and n-type doping. J. Mater. Chem. C.

[61] Rafols-Ribe J, Gracia-Espino E, Jenatsch S, Lundberg P, Sandstrom A, Tang S, Larsen C, Edman L (2021). Elucidating deviating temperature behavior of organic light-emitting diodes and light-emitting electrochemical cells. Adv. Opt. Mater..

[62] Fresta E, Dosso J, Cabanillas-Gonzalez J, Bonifazi D, Costa RD (2020). Revealing the impact of heat generation using nanographene-based light-emitting electrochemical cells. ACS Appl. Mater. Inter..

[63] Ràfols-Ribé J, Robinson ND, Larsen C, Tang S, Top M, Sandström A, Edman L (2020). Self-heating in light-emitting electrochemical cells. Adv. Funct. Mater..

[64] Diethelm M, Grossmann Q, Schiller A, Knapp E, Jenatsch S, Kawecki M, Nüesch F, Hany R (2019). Optimized electrolyte loading and active film thickness for sandwich polymer light-emitting electrochemical cells. Adv. Opt. Mater..

