# Supplementary material for: Chemical doping to control the in-situ formed doping structure in light-emitting electrochemical cells
Source: Sci Rep. 2023 Jul 15;13:11457. doi: 10.1038/s41598-023-38006-y (PMC10349809; doi:10.1038/s41598-023-38006-y)
Supplement: Supplementary file 1 — Supplementary Information. [file 41598_2023_38006_MOESM1_ESM.docx]

**Supporting Information:**

**Chemical Doping to Control the In-Situ Formed Doping Structure in Light-Emitting Electrochemical Cells**

*Gunel Huseynova,^1^ Joan Ràfols-Ribé,^1^ Etienne Auroux,^1^ Ping Huang,^2^ Shi Tang,^1^ Christian Larsen^1^ and Ludvig Edman^1,^**

^1^ The Organic Photonics and Electronics Group, Department of Physics, Umeå University, SE-90187 Umeå, Sweden

^2^ Department of Chemistry – Ångström Laboratory, Uppsala University, Box 523, 751 20 Uppsala, Sweden

*Corresponding author e-mail: ludvig.edman@umu.se


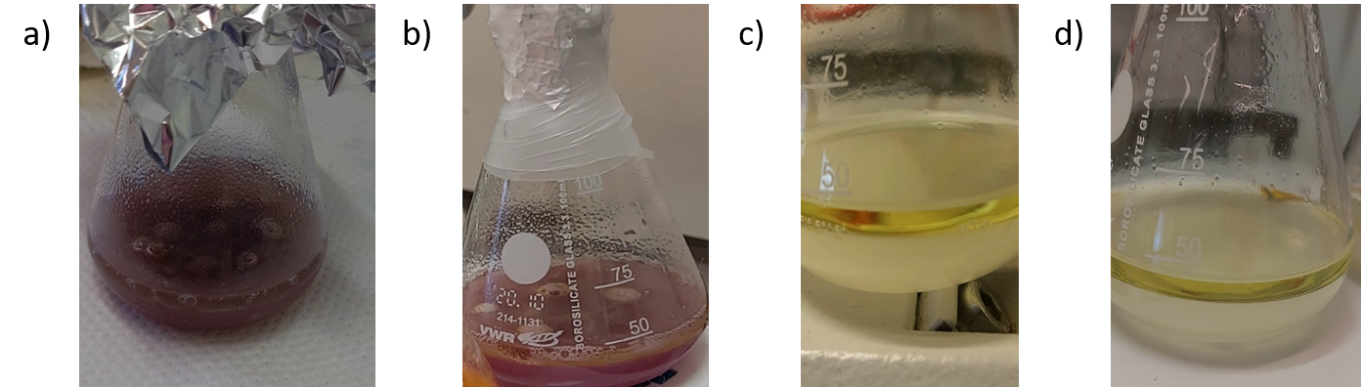

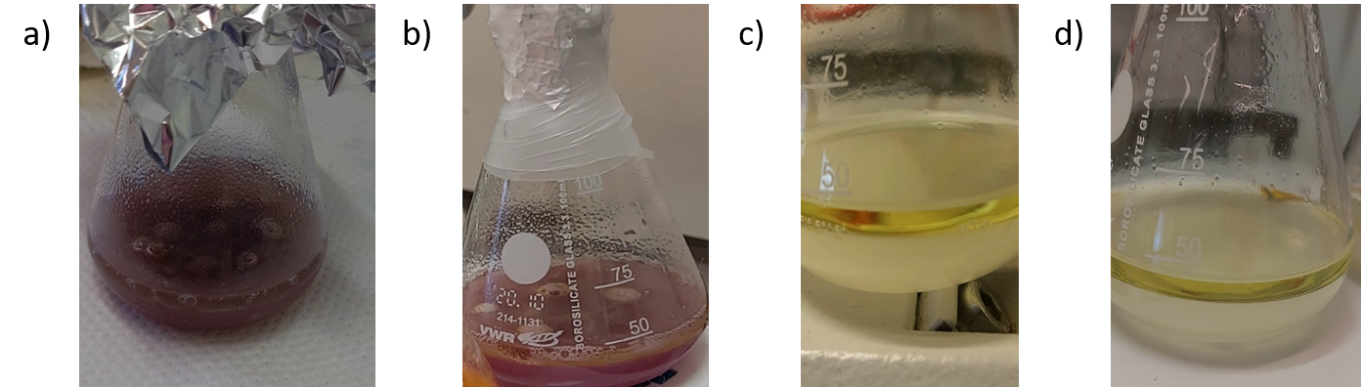


***Figure S1.*** *Photographs of the characteristic color transformation that takes place in the reaction solution during the synthesis of r-BV^0^. The originally transparent solution, comprising benzyl viologen dichloride, BV^2+^Cl^-^_2_, and NaBH_4_ in a mixture of deionized water and toluene, is first transforming into a violet color when r-BV^+^ forms (left photograph), and thereafter gradually shifting to a yellow color (right photograph) when the final r-BV^0^ product forms. Note, that the two toluene and water solvents are visibly separated in the right photograph, and that the neutral (yellow) r-BV^0^ product is solely dissolved in the lower-density and more hydrophobic toluene floating on top of the water.*


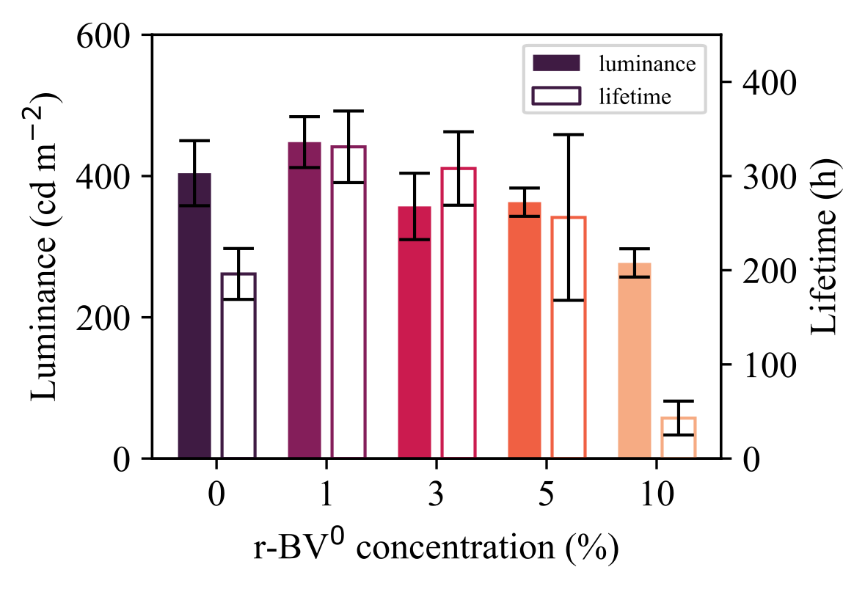


***Figure S2.*** *The average values for the peak luminance (solid bars, left axis) and the operational lifetime (open bars, right axis) as a function of the r-BV0 concentration. Each value is the average of ~8 devices and the error bars correspond to one standard deviation.*

***Table S1.*** *The average* LEC device metrics as a function of the r-BV^0^ concentration. The external quantum efficiency (EQE) was calculated assuming Lambertian emission.*

| **r-BV^0^ concentration**  **(mass%)** | **Peak luminance**  **(cd·m^-2^)** | **Minimum voltage (V)** | **Peak current efficacy (cd·A^-1^)** | **External quantum efficiency (%)** | **Peak power efficacy (lm·W^-1^)** | **Operational lifetime (>100 cd·m^-2^) (h)** |
| --- | --- | --- | --- | --- | --- | --- |
| 0 | 404±46 | 4.0±1.1 | 5.2±0.6 | 1.5±0.2 | 4.1±0.6 | 196±27 |
| 1 | 448±36 | 4.7±0.9 | 5.8±0.5 | 1.7±0.2 | 4.1±0.9 | 331±38 |
| 3 | 357±47 | 3.7±0.7 | 4.6±0.6 | 1.4±0.2 | 4.2±1.2 | 308±39 |
| 5 | 363±20 | 4.5±0.5 | 4.6±0.3 | 1.4±0.1 | 3.4±0.4 | 256±88 |
| 10 | 277±20 | 5.2±0.1 | 3.5±0.3 | 1.0±0.1 | 2.0±0.3 | 43±18 |

*The presented data are the average and standard deviation of ∼8 devices.

**Table S2.** Parameter selection for the drift-diffusion simulations.

| Parameter | Value | Unit |
| --- | --- | --- |
| Active-material thickness, *d*_AM_ | 150 | nm |
| Rel. permittivity | 2.8 |  |
| Mobility model | Constant mobility |  |
| Electron mobility, $\boldsymbol{\mu}_{\boldsymbol{n}}$ | 1.25×10^-5^ | cm^2^ V^-1^ s^-1^ |
| Hole mobility, $\boldsymbol{\mu}_{\boldsymbol{p}}$ | 4×10^-6^ | cm^2^ V^-1^ s^-1^ |
| Ionic mobility, $\boldsymbol{\mu}_{\boldsymbol{a,c}}$ | 10^-10^ | cm^2^ V^-1^ s^-1^ |
| HOMO/LUMO levels | 5.2/2.6 | eV |
| Injection model | Ohmic |  |
| Injection barrier | 0.1 | eV |
| Density of states DOS | 10^21^ | cm^-3^ |
| Ion concentration | 10^19^ | cm^-3^ |
| Applied voltage | 3.0 | V |
| Electron trap concentration | 8×10^17^– 8×10^18^ | cm^-3^ |


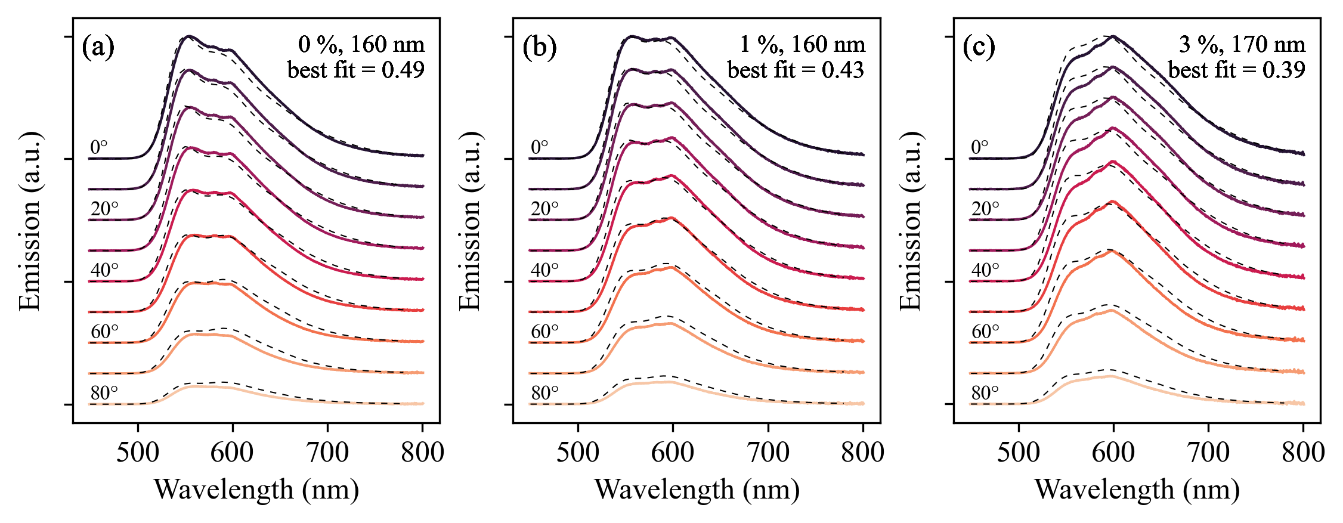


***Figure S3.*** *The measured (solid lines) and the best-fit simulated (dashed lines) EL spectra for a set of viewing angles (ranging between 0° and 80°) for the following r-BV^0^ concentrations: (a) [r-BV0] = 0 %, (b) [r-BV0] = 1 %, and (c) [r-BV0] = 3 %. The r-BV^0^ concentration, the thickness of the active material, and the best-fit value for the position of the p-n junction are identified in the upper right insets. The data were recorded at steady state.*


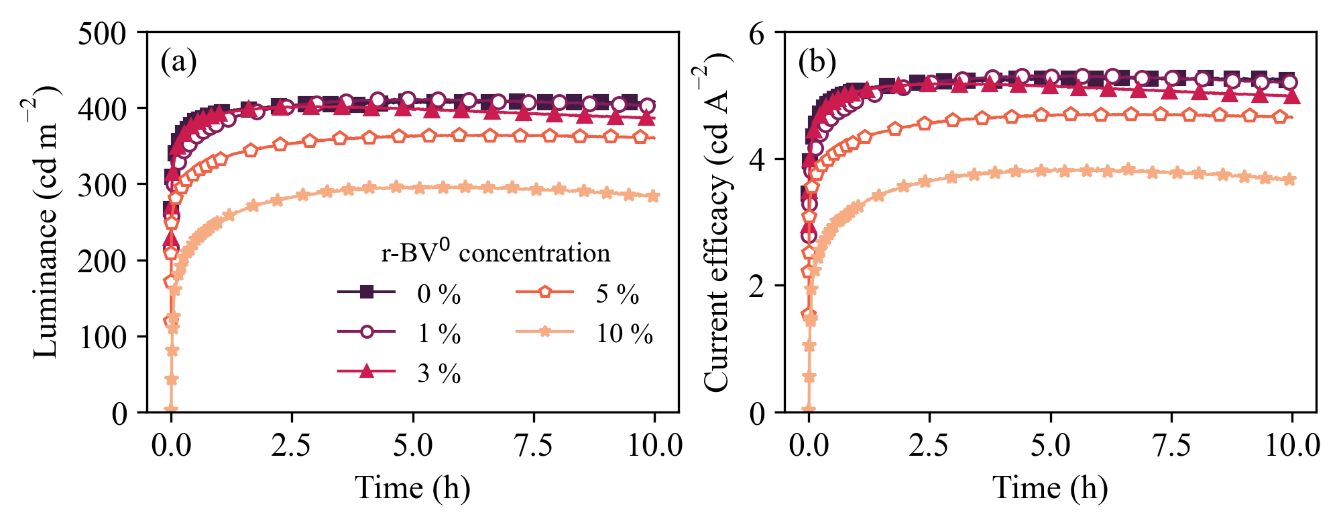


***Figure S4.*** *A close-up of the first ten hours of operation of the device data presented in Fig. 3d, showing (a) the luminance transient and (b) the current-efficacy transient. The concentration of the chemical reductant r-BV^0^ is identified in the inset in (a).*
